# Changes in PD-L1 expression according to tumor infiltrating lymphocytes of acquired EGFR-TKI resistant EGFR-mutant non-small-cell lung cancer

**DOI:** 10.18632/oncotarget.22582

**Published:** 2017-11-21

**Authors:** Tae-Jung Kim, Soon Auck Hong, Okran Kim, Seung Joon Kim, Ji-Hyun Yang, Eun Kyo Joung, Jin-Hyoung Kang, Sook-Hee Hong

**Affiliations:** ^1^ Department of Hospital Pathology, College of Medicine, The Catholic University of Korea, Seoul, Korea; ^2^ Department of Pathology, Soonchunhyang University Cheonan Hospital, College of Medicine, Soonchunhyang University, Cheonan, Republic of Korea; ^3^ Cancer Research Institute, The Catholic University of Korea, Seoul, Korea; ^4^ Division of Respiratory and Critical Care Medicine, Department of Internal Medicine, College of Medicine, The Catholic University of Korea, Seoul, Korea; ^5^ Division of Medical Oncology, Department of Internal Medicine, College of Medicine, The Catholic University of Korea, Seoul, Korea

**Keywords:** epidermal growth factor receptor, programmed death receptor ligand 1, CD8^+^ tumor infiltrating lymphocyte, non-small cell lung cancer

## Abstract

**Backgrounds:**

EGFR-mutant non-small cell lung cancer (NSCLC) that developed acquired resistance to EGFR-tyrosine kinase (TKI) are potential candidates for programmed death 1 (PD1) inhibitor.

**Results:**

TPS≥1% for PD-L1 and low CD8**^+^** TIL in post-TKI tumor showed a trend for a lower PFS of EGFR-TKIs (14.2 *vs* 9.9 months; *P* = 0.060) (cohort A). Only 2 of 22 specimens (9.1%) with an acquired EGFR exon 20 T790M mutation exhibited in post-TKI TPS≥50% for PD-L1. The degree in post-TKI tumor of PD-L1 expression was varied in 19 patients (40.5%), with 10 (21.2%) showing higher levels in the resistant biopsy (cohort B). Among the post-TKI high TPS groups, median PFS with low post- TKI CD8**^+^** TIL scores treated with EGFR-TKIs (6.6 months) was significantly lower than that for the other patients (14.2 months; *P* = 0.015).

**Conclusions:**

The change of PD-L1 expression was accompanied by dynamic change in CD8**^+^** TILs and might reflect diverse mechanism of resistance to EGFR-TKI therapy.

**Material and Methods:**

We identified 69 patients (cohort A) with sufficient post-TKI tumor tissues and 47 patients (cohort B) with paired tumor tissues available. TPS for PD-L1 expression of tumor cells and CD8**^+^** TILs score in tumor specimens were determined by immunohistochemistry.

## INTRODUCTION

Activating mutants of epidermal growth factor receptor (EGFR) are found in 40% of non-small cell lung cancer (NSCLC) patients in Asia and 10% of patients in Western countries [1]. Treatment outcome in EGFR-mutant NSCLC has been shown to outperform that in other lung cancers owing to the efficacy of EGFR tyrosine kinase inhibitors (EGFR-TKIs) [2, 3]. However, in most cases, resistance to EGFR-TKIs inevitably develops in 1 year [4]. Resistance to third-generation TKIs, which target the resistance mutation T790M in exon 20 acquired in response to first-generation EGFR-TKIs, also inevitably develops within 8–10 months [5, 6]. These observations highlight the need to develop new treatment strategies, including immunotherapeutics.

Monoclonal antibodies targeting the programmed death 1 (PD1) receptor and its ligand (PD-L1) have recently demonstrated promising antitumor efficacy in NSCLC [7, 8]. As a first-line therapy, the PD1 inhibitor pembrolizumab has shown significant improvement in overall survival compared with standard platinum doublet chemotherapy in NSCLC with high PD-L1 expression [9]. However, treatment outcomes following immunotherapy was reported to be less effective in patients with EGFR-mutant NSCLC than in those with EGFR wild type NSCLC, based on subgroup analyses of Phase III clinical trials [10]. To date, increased PD-L1 expression has been associated with greater benefit of PD1 inhibitor therapy [8, 11]. However, low expression of both PD-L1 and CD8-positive (CD8^+^) tumor-infiltrating lymphocytes (TILs) in advanced EGFR-mutant NSCLC was reported to underlie the low overall response rate to PD1 inhibitors [12, 13]. In addition, tumor mutation burden and smoking exposure are also considered potential biomarkers supporting PD1 inhibitor treatment [14]. Generally, EGFR-mutant NSCLCs that develop in those who never smoked tend to have less of a mutation burden than NSCLCs in smokers. However, patients with EGFR-TKI resistant EGFR-mutant NSCLC with high tumor PD-L1 expression, but without the EGFR exon 20 T790M mutation, have shown favorable efficacy in nivolumab therapy [15].

Patients with EGFR-mutant NSCLC that have acquired resistance to EGFR-TKI are potential candidates for PD1 inhibitor treatment. Because diverse EGFR-TKI resistance mechanisms, including crosstalk between the tumor and its microenvironment, have been reported, PD-L1 expression and TIL status before developing acquired resistance to EGFR-TKIs may not accurately reflect the status after developing acquired resistance to EGFR-TKIs. Here, we retrospectively analyzed the tumor proportion score (TPS) for PD-L1 expression and CD8^+^ TILs in paired tumor tissues from NSCLC patients harboring activating EGFR mutants before and after developing acquired resistance to EGFR-TKIs. In addition, we explored changes in PD-L1 expression and TILs in relation to clinical outcome following EGFR-TKI therapy and acquisition of EGFR exon 20 T790M mutations.

## RESULTS

### Patient characteristics

A total of 69 eligible patients with advanced NSCLC were included in the present study (Table 1). The mean age at diagnosis was 60.2 years (range, 34–78.4 years). Fifty of the 69 patients (72.5%) were female and 15 (21.7%) were smokers. With regard to EGFR mutation status, 49 patients harbored a deletion in exon 19, and 18 patients had an L858R/L861Q missense mutation in exon 21, two of which were uncommon mutations (G719X in exon 18 and S768I in exon 20). Of the 62 patients who underwent EGFR mutation testing after developing acquired resistance to first or second generation EGFR-TKIs, 22 carried the exon 20 T790M mutation. Fifty seven patients were treated with EGFR-TKIs as a first-line therapy and 12 patients were treated with EGFR-TKIs as a second-line therapy. The median PFS for was 13.4 months for first-line EGFR-TKI therapy and 20.4 months for second-line EGFR-TKI therapy.

**Table 1:** Clinical and pathologic characteristics of patients with EGFR mutant NSCLC before and after EGFR-TKI therapy

Characteristics	*N* = 69
Age (mean, Years)	60.2 (34–78.4)
Sex	
Men	19 (27.5%)
Women	50 (72.5%)
ECOG Performance status	
0	47 (68.1%)
1	18 (26.1%)
2	4 (5.8%)
Smoking history	
Current or ex-smoker	15 (21.7%)
Never smoker	54 (78.3%)
Clinical stage at diagnosis	
Stage IIIB	5 (7.2%)
Stage IVa	19 (27.5%)
Stage IVb	45 (65.2%)
Presence of brain metastasis	24 (34.7%)
Histology	
Adenocarcinoma	66 (95.7%)
Adenosquamous cell carcinoma	2 (2.9%)
Squamous cell carcinoma	1 (1.4%)
EGFR mutation at diagnosis	
Exon 19 del	49 (71.1%)
Exon 21 L858R/L861Q	18 (26.1%)
Exon 18 G719X	1 (1.4%)
Exon 20 S768I	1 (1.4%)
Rebiopsy histology	
Adenocarcinoma	59 (85.6%)
Small cell carcinoma/neuroendocrine carcinoma	4 (5.8%)
Adenosquamous/Squamous cell carcinoma	4 (5.8%)
Large cell carcinoma	1 (1.4%
Sarcomatoid carcinoma	1 (1.4%)
Acquired EGFR Exon 20 T790M mutation in rebiopsy	
Exon20 T790M	22 (31.9%)
No exon20 T790M	40 (58.0%)
Unknown	7 (10.1%)
Treatment just before rebiopsy	
EGFR-TKIs	57 (82.6%)
Cytotoxic chemotherapy	12 (17.4%)
Median PFS for EGFR-TKIs	Median Months (95% CI)
First line (*n* = 53)	13.4 (11.7–15.0)
Second line (*n* = 16)	20.4 (8.7–32.5)

### PD-L1 expression and CD8^+^ TILs in EGFR-mutant NSCLCs after acquired resistance to EGFR-TKIs

**Figure 1:**
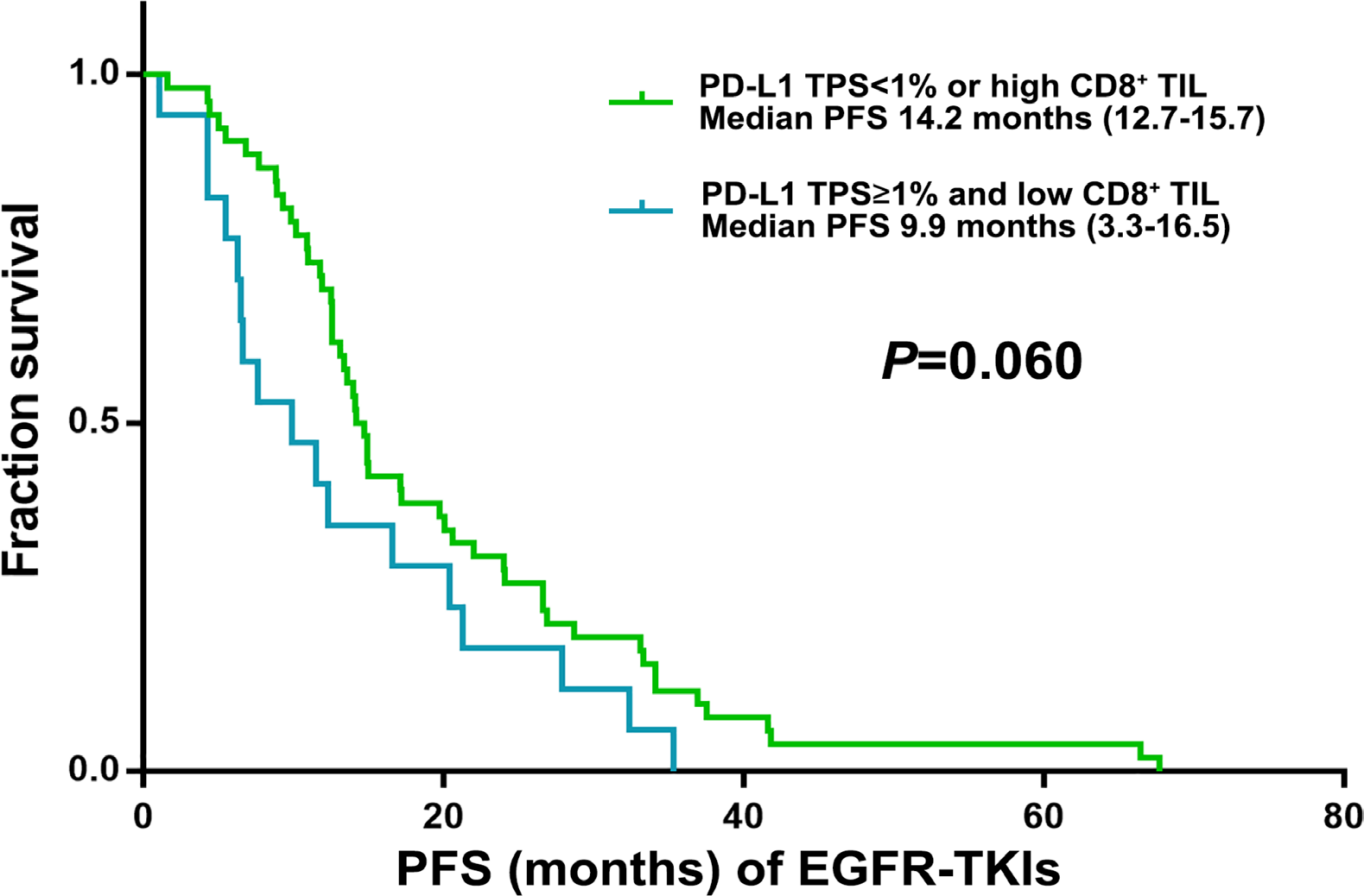
Kaplan–Meier curves for PFS of EGFR-TKI according to post-EGFRT-TKI TPS for PD-L1 and CD8^+^ TILs score (*n* = 69)

PD-L1 expression was detected in 21 (30.4%) and 11 (15.9%) of 69 post-EGFR-TKI specimens using cut-offs of 1–49% and ≥50% in tumor proportion score (TPS; see Methods), respectively (Table 2). We also observed high-level (grade ≥2) CD8^+^ TILs in 12 (57.2%) and 3 (27.3%) in 1–49% and TPS≥50% group, respectively (Table 2). TPS≥1% for PD-L1 and low CD8^+^ TIL in post-EGFR-TKI specimens showed a trend for a lower PFS of EGFR-TKIs (9.9 *vs* 14.2 months; *P* = 0.060) (Figure 1). The other clinical characteristics were not significantly different (Table 3). 4 of 22 specimens (18.2%) with an acquired EGFR exon 20 T790M mutation exhibited in TPS≥1% for PD-L1 and low CD8^+^ TIL.

**Table 2:** Tumor proportion score (TPS) for PD-L1 and CD8^+^ tumor infiltrating lymphocyte (TIL) score after EGFR-TKI therapy (*n* = 69)

Post-TKI PD-L1		CD8^+^ TIL score		*P* value
Tumor proportion score	No. of patients	TIL score	No. of patients	
0%	37	0/1+	21 (56.7%)	0.286
	(53.6%)	2+/3+	16 (43.3%)	
1–49%	21	0/1+	9 (42.8%)	
	(30.4%)	2+/3+	12 (57.2%)	
≥50%	11	0/1+	8 (72.7%)	
	(15.9%)	2+/3	3 (27.3%)	

**Table 3:** Clinical characteristics according to post-EGFR-TKI TPS for PD-L1 and CD8^+^ TIL (*n* = 69)

TPS for PD-L1/CD8^+^ TIL score	PD-L1 TPS< 1% or high CD8^+^TIL (*n* = 52)	PD-L1TPS≥ 1% and low CD8^+^ TIL (*n* = 17)	*P* value
M/F	14/38	5/12	1.0
Smoking			0.410
Current or ex-smoker	11 (21.2%)	4 (23.5%)	
Never smoker	41 (78.8%)	13 (76.5%)	
Treatment before rebiopsy			0.472
EGFR TKI	44 (86.4%)	13 (76.5%)	
Cytotoxic chemo	8 (15.4%)	4 (23.5%)	
Age (mean, Years)	62.3	61.5	0.771
EGFR mutation at diagnosis			0.163
Exon19	36 (69.2%)	13 (76.5%)	
Exon 21 and others	16 (30.8%)	4 (23.5%)	
PFS (Mo) of EGFR-TKI	14.2 (12.7–15.7)	9.9 (3.3–16.5)	0.060
EGFR Exon 20 T790M mutation after EGFR-TKI			
Exon20T790M	18 (34.6%)	4 (23.5%)	0.403
No exon20 T790M	34 (65.4%)	13 (76.5%)	
Stage			0.384
IIIB/IVA	16 (30.7%)	8 (47.1%)	
IVB	36 (69.3%)	9 (52.9%)	
Presence of brain metastasis	18 (34.6%)	6 (35.3%)	0.959

### Dynamic changes in PD-L1 expression and CD8^+^ TILs after EGFR-TKI therapy

**Figure 2:**
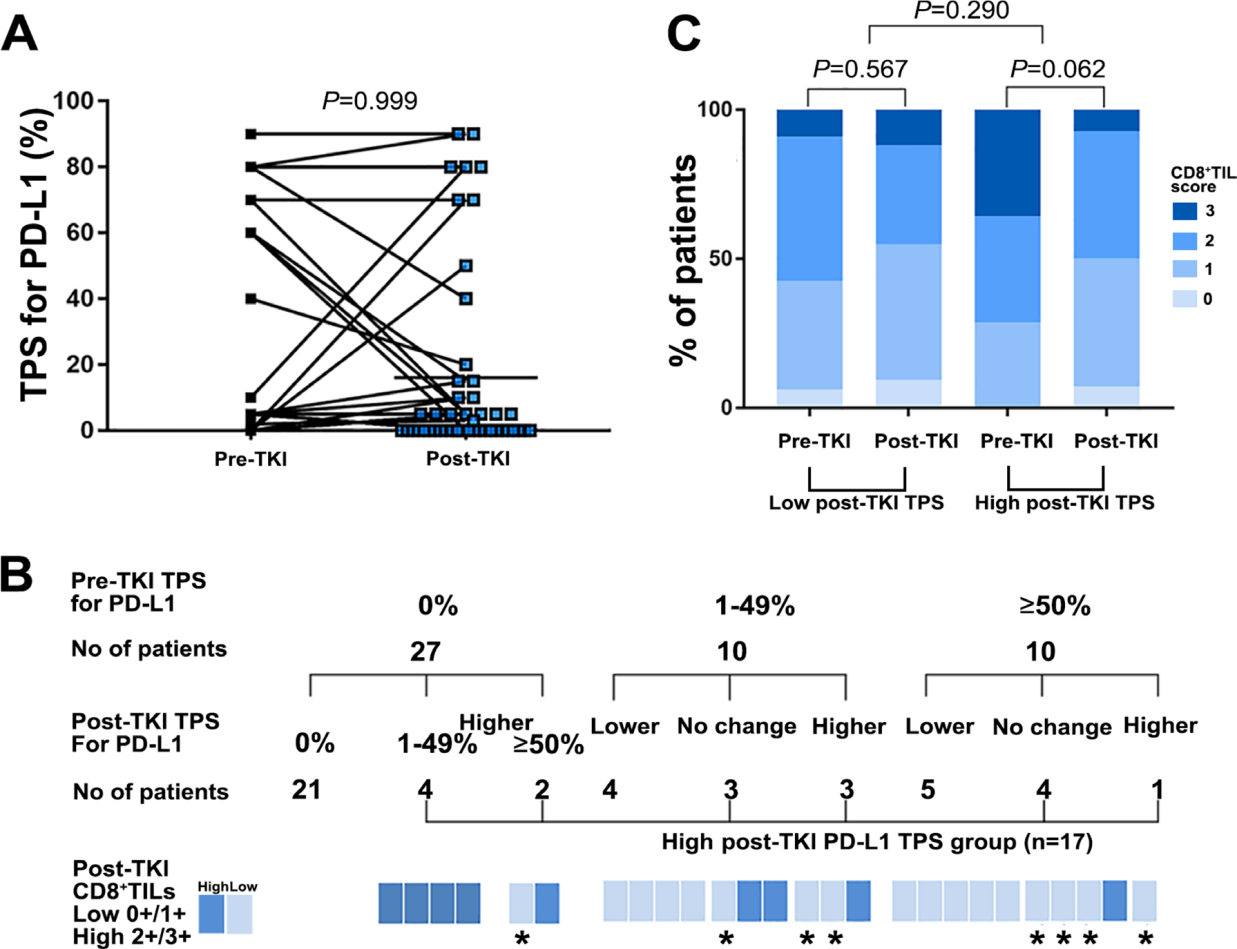
PD-L1 expression and CD8^+^ TILs before and after developing acquired resistance to EGFR-TKI therapy among patients with available paired tissues (*n* = 47) (**A**) Change of TPS for PD-L1in EGFR mutant NSCLC before EGFR-TKI and after developing resistance to EGFR-TKI among the patient with available paired biopsies (*n* = 47). (**B**) Change of TPS for PD-L1 according to pre-TKI TPS for PD-L1 among the patient with available paired biopsies (*n* = 47). ^*^High post -TKI TPS for PD-L1 and post-TKI low CD8^+^ TILs score patients (*n* = 8). (**C**) Change of CD8^+^ TILs score in EGFR mutant NSCLC pre-TKI and post-TKI between high and low post-TKI TPS of PDL-1 group among the patient with available paired biopsies (*n* = 47).

To determine whether targeted therapies affected PD-L1 expression and infiltration of CD8^+^ lymphocytes, we analyzed the TPS for PD-L1 expression and CD8^+^ TILs in paired tumor biopsies obtained from patients before starting EGFR-TKIs and after developing resistance to EGFR-TKIs (*n* = 47, cohort B). Comparisons of paired pre- and post-EGFR-TKI biopsies showed that the degree of PD-L1 expression was not changed in both biopsies in 28 patients (59.6%), but varied upon the development of resistance in 19 patients (40.4%), with 10 showing higher levels of PD-L1 expression in the resistant biopsy (*P* = 0.999; Figure 2A and 2B). Twenty one of 27 patients (77.8%) with a TPS for PD-L1 of 0% before EGFR-TKI were also 0% after EGFR-TKI therapy. The remaining 6 patients showed an increased TPS for PD-L1 expression after EGFR-TKI (Figure 2B). Of these six patients, five also had a high post-EGFR-TKI CD8^+^ TIL score. In seven of ten patients with a pre-EGFR-TKI TPS for PD-L1 expression in the 1–49% range, the TPS for PD-L1 changed post-EGFR-TKI as followed; one of three patients with increased TPS for PD-L1 exhibited an high CD8^+^ TIL score post-EGFR-TKI, whereas all of four with decreased TPS for PD-L1 showed a low post-EGFR-TKI CD8^+^ TIL score (Figure 2B). Of the ten patients with a pre-EGFR-TKI TPS≥50%, the post-EGFR-TKI TPS for five was as high or higher than the pre-EGFR-TKI TPS; four of these five patients had a low post-EGFR-TKI CD8^+^ TIL score (Figure 2B).

We also observed high-level (grade ≥ 2) CD8^+^ TILs in 29 (61.7%) pre-EGFR-TKI specimens and 22 (46.8%) post-EGFR-TKI specimens. The CD8^+^ TIL score was consistent between paired pre- and post-EGFR-TKI biopsies in 15 patients (31.9%), but changed upon the development of resistance in 32 patients (68.1%), with 18 (38.3%) showing a higher CD8^+^ TIL score in the resistant biopsy.

### Clinical characteristics of patients with high TPS for PD-L1 expression in resistant biopsies

To determine the relationship between post-EGFR-TKI TPS for PD-L1 expression and clinical characteristics, we defined a post-EGFR-TKI high TPS group for PD-L1 expression who observed higher TPS for PD-L1 in post-EGFR-TKI biopsies than paired pre-EGFR-TKI biopsies or not decreased post-EGFR-TKI TPS for PD-L1 ≥ 1% compared to pre-EGFR-TKI biopsies (*n* = 17, Figure 2B).

Patients in the high post-EGFR-TKI TPS group for PD-L1 expression tended to have a higher pre-TKI CD8^+^ TIL score than those in the group with a low TPS for PD-L1 expression after EGFR-TKI therapy. However, post-TKI CD8^+^ TIL scores in the high post-EGFR-TKI TPS for PD-L1 group decreased after developing acquired resistance to EGFR-TKI therapy (*P* = 0.062) compared with that observed in the group with low post-EGFR-TKI TPS, which did not significantly change (*P* = 0.290; Figure 2C).

**Figure 3:**
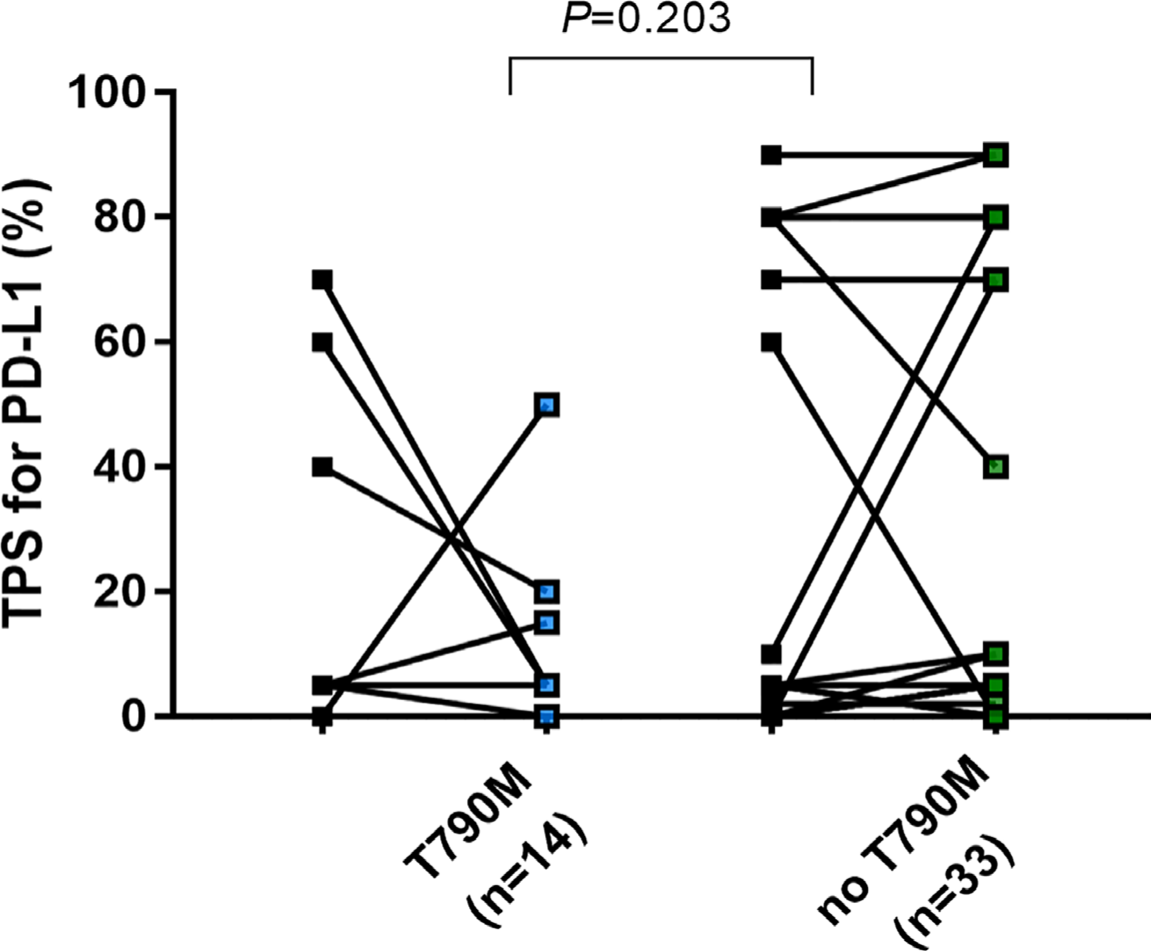
Change of TPS of PD-L1in EGFR mutant NSCLC before EGFR-TKIs and after developing acquired resistance to EGFR-TKIs based on presence of acquired exon20 T790M mutation

Only 2 of 14 specimens (14.2%) with an acquired EGFR exon 20 T790M mutation exhibited a high TPS group for PD-L1 expression in post-EGFR-TKI biopsies (Figure 3).

**Figure 4:**
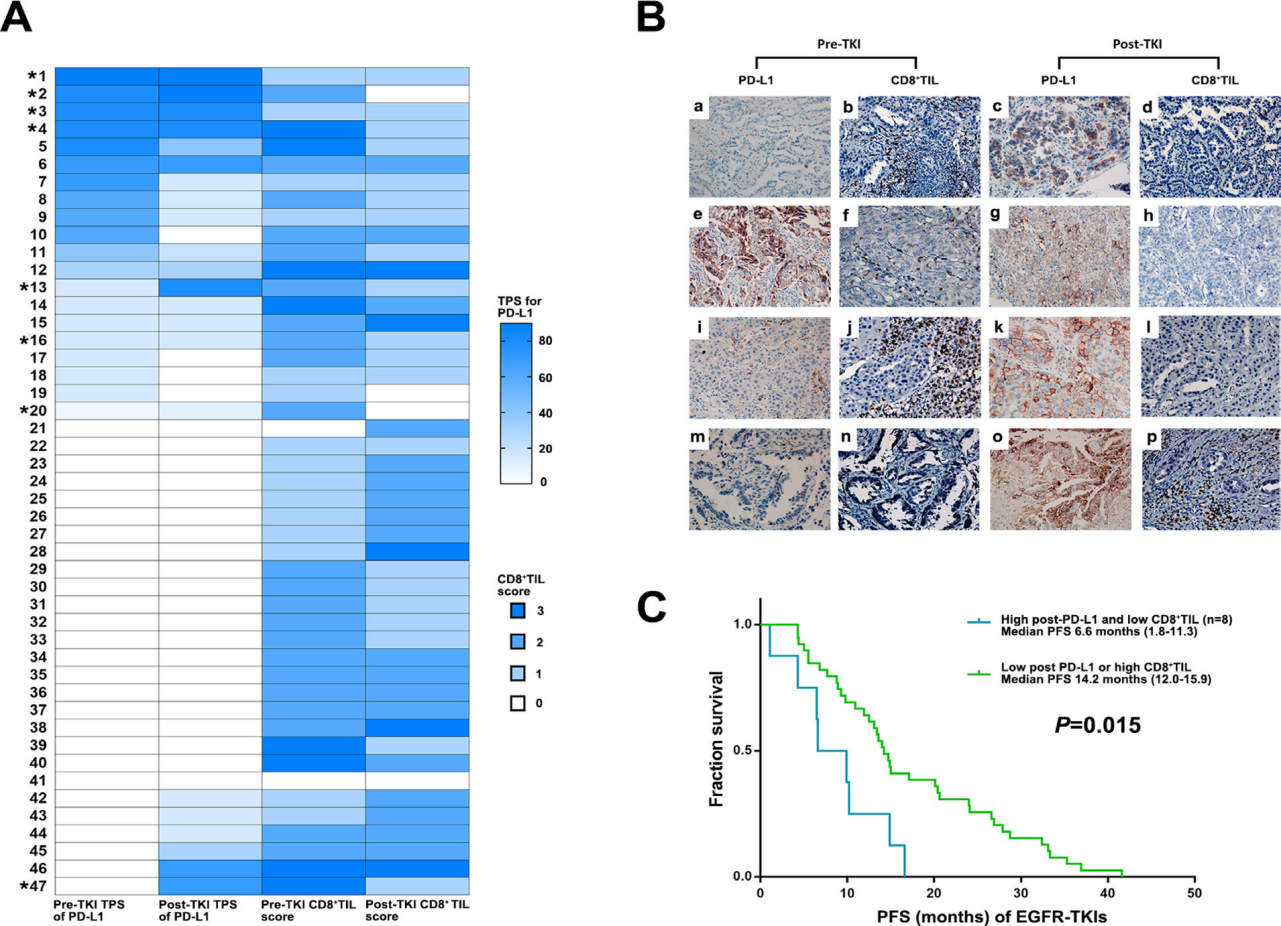
Clinical outcome of EGFR TKIs according to dynamic changes in PD-L1 expression and CD8^+^ TILs score (**A**) Change of TPS for PD-L1 and CD8^+^ TILs in EGFR mutant NSCLC before EGFR-TKIs and after developing acquired resistance to EGFR-TKIs among the patient with available paired biopsies (^*^8 patients with high post-TKI TPS for PD-L1 and low CD8^+^ TIL). (**B**) PD-L1 expression and CD8^+^ TILs before and after EGFR- TKIs in paired biopsies with represesntive immunohitochemical images. Pre-TKI with (a) no PD-L1 expression and (b) high CD8^+^ TIL showed post-TKI with (c) high PD-L1 (70%) and (d) low CD8+ TIL (No. 47 in Figure 4A). Pre-TKI with (e) high PD-L1 expression (80%) and (f) high CD8^+^ TIL showed post-TKI with (g) high PD-L1 expression (90%) and (h) low CD8^+^ TIL (No. 2 in Figure 4A). Pre-TKI with (i) low PD-L1 expression (10%) and (j) high CD8^+^ TIL showed post-TKI with (k) high PD-L1 (80%) and (l) low CD8^+^ TIL (No. 13 in Figure 4A). Pre-TKI with (m) no PD-L1 expression and (n) low CD8^+^ TIL showed post-TKI with (o) high PD-L1 (70%) and (p) high CD8+ TIL (No. 43 in Figure 4A). (**C**) Kaplan–Meier curves for PFS of EGFR-TKIs according to post-EGFRT-TKI PD-L1 TPS and CD8^+^ TILs (Log-rank test).

Based on the finding of decreased post-EGFR-TKI CD8^+^ TILs after developing resistance to EGFR-TKIs in the high post-EGFR-TKI TPS group for PD-L1, we individually analyzed TPS for PD-L1 expression and CD8^+^ TILs. This analysis showed that 8 of 17 patients in the high post-EGFR-TKI TPS group for PD-L1exhibited low post-EGFR-TKI CD8^+^ TIL scores (Figure 4A and 4B). The median PFS for the eight patients in this group treated with first EGFR-TKIs (6.6 months) was significantly lower than that for the other patients (14.0 months; *P* = 0.015) (Figure 4C).

## DISCUSSION

In this retrospective study, we evaluated changes in PD-L1 expression and CD8^+^ TILs after developing acquired resistance to EGFR TKI therapy. Gainor *et al*. recently reported that the expression of PD-L1 was changed in 28% of patients after EGFR-TKI therapy; Han et al. also reported a marked increase (38.9%) in the expression of PD-L1 [12, 16]. Our study showed that the expression of PD-L1 was changed in 40.9% of patients, with increasing in 10 (22.7%) of them. Thus, similar to previous studies, our study showed that the expression of PD-L1 changed after EGFR-TKI therapy. Because heterogeneity in PD-L1 expression after EGFR-TKI therapy we analyzed whether changes in PD-L1 expression were related to CD8^+^ TIL score. Among 10 patients with increased post-TKI TPS for PD-L1 expression, six showed high in post-TKI CD8^+^ TIL score and the other four showed low. Notably, five of ten patients with pre-EGFR-TKI TPS for PD-L1 ≥ 50% showed greater than 50% post-TKI TPS and four of them showed a low post-EGFR-TKI CD8^+^ TIL score. In contrast, among 6 of 27 patients (22.2%) with increased post-EGFR TKI TPS for PD-L1 from pre-EGFR-TKI TPS for PD-L1 0%, five patients a high post-EGFR-TKI CD8^+^ TIL score. That means high post-EGFR-TKI TPS for PD-L1 group tended to decrease post-TKICD8^+^ TIL score. The PD-L1 expression of this high post-TKI TPS for PD-L1 group may not be related CD8^+^ TIL mediated IFN-gamma signaling [17].

*In vitro* studies of EGFR-mutant lung cancer have shown that the expression of PD-L1 is increased in these cancer cells through downstream effectors in the EGFR pathway, such as STAT3 (signal transducer and activator of transcription 3) and ERK1/2 (extracellular signal-regulated kinase 1/2) [18–20]. These studies also showed that inhibition of the EGFR pathway reduced the expression of PD-L1, suggesting that the oncogenic EGFR pathway is the main mechanism regulating PD-L1 expression in EGFR-mutant lung cancer [18–20]. PD-L1 expression was also increased by the EGFR-TKI–resistant oncogenic pathway after acquisition of resistance to EGFR-TKI [16, 20]. Han et al. reported that PD-L1 expression was increased in tumors exhibiting epithelial mesenchymal transition (EMT) upon developing resistance to EGFR-TKI [16]. Further studies are needed to determine whether an increase in PD-L1 leads directly to EGFR-TKI resistance or is one of the secondary changes in the EGFR-TKI resistance mechanism, such as EMT and amplification of the c-MET or MEK-ERK pathway.

Our data suggest that cases of high post-EGFR-TKI PD-L1 expression and low CD8^+^ TILs could be related to innate immune resistance caused by activation of the EGFR-TKI resistant oncogenic pathway [21]. Thus, in the case of higher PD-L1 expression and low CD8^+^ TIL post-EGFR-TKI, the duration of tumor control by EGFR-TKI therapy decreased. Also, because high CD8^+^ TIL is also known to be a predictive indicator for the use of PD1 inhibitors, the low post-EGFR-TKI CD8^+^ TIL suggests that PD1 inhibitors might not be effective even though high PD-L1 expression [13].

Our data suggest that, in patients with both high PD-L1 expression and a high CD8^+^ TIL score post EGFR-TKI treatment, the expression of PD-L1 might be related to acquired immune resistance through TILs [17]. Consistent with that infiltration of CD8^+^ lymphocytes is an important predictive marker for the efficacy of immunotherapeutic agents, such as those targeting the PD1/PD-L1 pathway, PD1 inhibitor therapy was effective in patients with both increased post-EGFR-TKI PD-L1 expression and CD8^+^ TIL scores compared with patients exhibiting increased post-EGFR-TKI expression of PD-L1 alone [13]. Haratani et al. recently demonstrated that nivolumab treatment was highly effective against exon 20 T790M negative EGFR-TKI–resistant EGFR-mutant NSCLCs in cases where the EGFR-TKI–resistant tumor expressed high levels of PD-L1 together with high CD8^+^ TIL [15]. They also reported that the proportion of tumors with high expression of PD-L1 was greater among T790M–negative patients than among T790M–positive patients [15]. Our study also showed an increase in post-EGFR-TKI expression of PD-L1 in only 2 of 14 patients harboring the EGFR exon 20 T790M mutation. It was recently reported that tumor mutation burden in EGFR-TKI–resistant EGFR-mutant NSCLCs might be an important predictive indicator for PD1 inhibitor immunotherapy [15]. EGFR exon 20 T790M mutant EGFR-TKI–resistant tumors were reported to have a lower mutation burden than other EGFR-TKI–resistant tumors [15].

Our study has several limitations. First, a small number of patients were included in the study, reflecting difficulties in obtaining appropriate paired biopsies before and after EGFR-TKI therapy. Second, analyses of PD-L1 expression and CD8^+^ TILs by immunohistochemical staining have not yet been standardized. For PD-L1 immunostaining, we used a relatively standardized scoring system based on TPS for PD-L1 employing a PharmDx kit; however, different PD-L1 scoring protocols have been used for different PD1 inhibitors. For scoring lymphocyte infiltration, we used a relatively standardized stromal CD8^+^ TIL scoring system, and to date there is no standardized CD8^+^ TIL scoring protocol for lung cancer [22]. The last practical limitation is related to dynamic changes in PD-L1 expression and CD8^+^ TILs. Because of the relative long-term survival of patients with EGFR-mutant NSCLC, the long-term effects of EGFR-TKI on tumors and the surrounding microenvironment, such as changes in phenotype, heterogeneity of resistance mechanisms, changes in the immune microenvironment and tumor-associated fibrosis, should be analyzed concurrently.

In conclusion, PD-L1 expression changed markedly after developing resistance to EGFR-TKIs. The results of our study suggest that neither post-EGFR-TKI PD-L1 expression nor CD8^+^ TIL score are related to pre-EGFR-TKI expression of PD-L1 or CD8^+^ TIL. These changes in PD-L1 expression were accompanied by dynamic changes in CD8^+^ TILs and might reflect diverse mechanism of resistance to EGFR-TKI therapy. Before considering PD1 inhibitor-based immunotherapy, PD-L1 expression and immune microenvironment status should be reevaluated.

## MATERIALS AND METHODS

We reviewed the medical records of all patients with advanced or recurrent EGFR-mutant NSCLC treated at Seoul St. Mary's Hospital, The Catholic University of Korea, between August 2010 and January 2017, identifying 101 patients who had undergone a rebiopsy or surgery after progression following EGFR-TKI therapy. Cytology specimens were excluded.

For analysis of PD-L1 expression, we identified 69 patients with sufficient archival post-TKI tumor tissue (cohort A) and 47 patients with sufficient paired archival tumor tissue (cohort B) available for analysis (Supplementary Figure 1). Formalin-fixed paraffin-embedded tissue was retrieved, and full sections were preferred over biopsies whenever possible. Whole slides were evaluated and he corresponding histology slides were reviewed for tissue adequacy (TJK and SAH). All studies were performed using Institutional Review Board (IRB)-approved protocols.

### Data collection

Clinicopathologic features and treatment histories were obtained by reviewing patients’ medical records. Data were updated as of June 2017. Responses were assessed according to Response Evaluation Criteria In Solid Tumor (RECIST, v1.1) [23]. Progression free survival (PFS) was measured from the time of treatment initiation to clinical/radiographic progression or death. Patients without documented clinical or radiographic disease progression were censored on the date of last follow-up.

### Immunohistochemistry

Consecutive 3 μm thick sections from the formalin-fixed, paraffin embedded (FFPE) non-small cell lung cancer (NSCLC) tissue were mounted on Dako FLEX IHC microscope slides, pretreated and stained with the PD-L1 antibody (clone 22C3) from pharmDx EnVision FLEX visualization system on Autostainer Link 48 (Agilent/Dako, Santa Clara, CA, USA) in accordance with the manufacturers’ instructions. PD-L1 staining is well validated and in rountine clinical use [24]. The other consecutive sections were stained with CD8 (1:100, clone C8/144B Agilent/Dako, Santa Clara, CA, USA) Briefly, the samples were heated in Tris-EDTA buffer (pH 9.0) at 95°C for 40 min. To block endogenous peroxidase activity, all the sections were treated with 100% methanol containing 0.3% H_2_O_2_ for 15 min. Nonspecific binding of IgG was blocked by using normal rabbit serum (Agilent/Dako). The sections were incubated with mouse anti-CD8+ monoclonal abs (Agilent/Dako) overnight at 4°C Then, they were incubated with biotinylated rabbit-anti-mouse secondary Abs (Agilent/DAKO) followed by the incubation with streptavidin-peroxidase complex solution for 30 min. Signals were generated by incubation with 3,3′-diaminobenzidine. Finally, the sections were counterstained with hematoxylin. Tonsil tissue was included as positive control for both low expression (macrophages in germinal centers) and high expression (crypt epithelium) for all assays. Controls provided with the pharmDx Dako 22C3 assays (sections from cellblocks with PD-L1 positive and negative cell lines) were also included in each run for these assays, in accordance with the manufacturers’ instructions. All slides were independently evaluated by two board certified pathologists (TJK, and SAH).

All viable tumor cells on the entire slide was evaluated and included in the PD-L1 scoring assessment. A minimum of 100 viable tumor cells present in the PD-L1 stained slide was considered adequate for PD-L1 evaluation. Partial or complete cell membrane staining (≥1+) that is perceived distinct from cytoplasmic staining. Cytoplasmic staining was considered non-specific staining and was excluded in the assessment of staining intensity. Normal cells and tumor associated immune cells such as infiltrating lymphocytes or macrophages was separately scored and was not included in the scoring for PD-L1 positivity. PD-L1 protein expression is determined by using Tumor Proportion Score (TPS), which is the percentage of viable tumor cells showing partial or complete membrane staining at any intensity. The specimen should be considered to have no expression (TPS<1%), low PD-L1 expression (TPS≥1%) and high PD-L1 expression (TPS≥50%) (Supplementary Figure 2) [24]. The degree of CD8^+^ TILs were semi-quantitatively evaluated on a scale of 0 to 3 based on the extent of positive lymphocyte infiltration in the stromal compartment (Supplementary Figure 2) [25]. Each score was defined the percentage of CD8^+^ T cells compared with total nucleated cells in the stromal compartment, as follows: score 0, <5%; score 1, 5% to 25%; score 2, 25% to 50%; score 3, >50% as previously described [25]. Scores were subsequently dichotomized into high (scores 2 and 3) and low (scores 0 and 1) for increased CD8^+^ TILs.

### Mutational analysis

Tumor specimens were obtained from each patient using diagnostic or surgical procedures. EGFR mutation testing was performed using nested polymerase chain reaction (PCR) and direct sequencing or a PNA-mediated PCR clamping method (PNAClamp EGFR Mutation Detection Kit; PANAGENE, Inc., Daejeon, Korea) by real-time PCR, as previously described.

### Statistical analysis

Fisher's exact test and the Wilcoxon rank sum test were applied to compare categorical variables and continuous variables, respectively. PFS was estimated by the Kaplan-Meier method using SPSS software (version 24.0; IBM Corp., Armonk, NY, USA). Differences in PFS between cohorts were assessed using a log rank test and were estimated as a hazards ratio (HR) using proportional hazards regression. Paired comparisons of PD-L1 expression and CD8^+^ TILs between pre- and post-EGFR-TKI specimens from the same patient were evaluated using McNemar's test (a marginal homogeneity test) and Wilcoxon sign-rank test, respectively, depending on whether the immunostaining data were reported as binary, ordinal, or continuous. All *P*-values are based on a two-sided hypothesis; calculations were performed using SPSS version 24.0 (IBM Corp.).

## SUPPLEMENTARY MATERIALS FIGURES


