# Supplementary material for: Changes in PD-L1 expression according to tumor infiltrating lymphocytes of acquired EGFR-TKI resistant EGFR-mutant non-small-cell lung cancer
Source: Oncotarget. 2017 Nov 21;8(64):107630–9. doi: 10.18632/oncotarget.22582 (PMC5746096; doi:10.18632/oncotarget.22582)
Supplement: Supplementary file 1 [file oncotarget-08-107630-s001.pdf]

Changes of PD-L1 expression according to tumor infiltrating lymphocytes in acquired EGFR-TKI resistant EGFR-mutant non-small-cell lung cancer

SUPPLEMENTARY MATERIALS

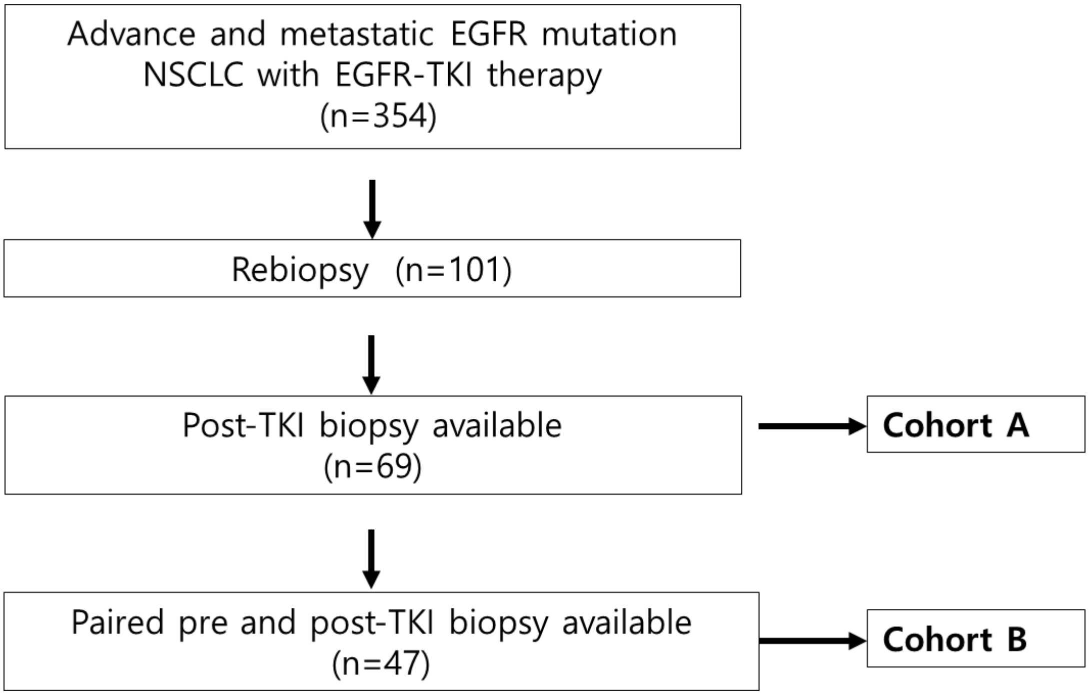

Supplementary Figure 1: Flowing chart of enrollment.

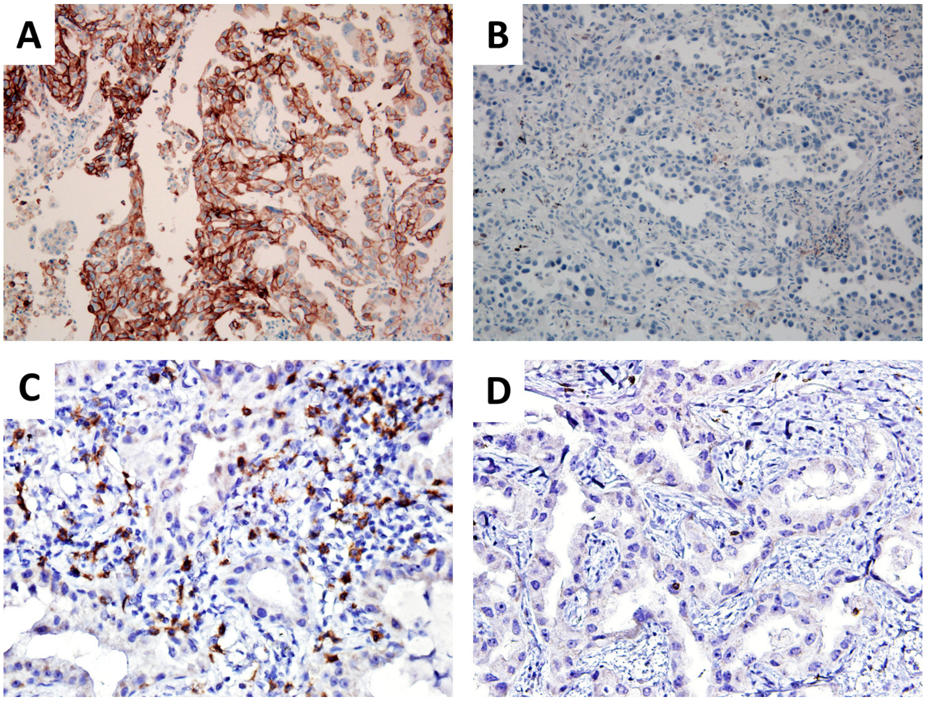

Supplementary Figure 2: Representative PD-L1 and CD8$^+$ tumor infiltrating lymphocyte (TIL) immunohistochemistry. Tumor with (**A**) high PD-L1 expression and (**B**) negative PD-L1 expression. TIL with (**C**) high CD8$^+$ score and (**D**) low CD8$^+$ score.
